# Assisted documentation as a new focus for artificial intelligence in endoscopy: the precedent of reliable withdrawal time and image reporting

**DOI:** 10.1055/a-2122-1671

**Published:** 2023-08-23

**Authors:** Thomas J. Lux, Zita Saßmannshausen, Ioannis Kafetzis, Philipp Sodmann, Katja Herold, Boban Sudarevic, Rüdiger Schmitz, Wolfram G. Zoller, Alexander Meining, Alexander Hann

**Affiliations:** 1 Interventional and Experimental Endoscopy (InExEn), Internal Medicine II, University Hospital Würzburg, Würzburg, Germany; 2 Department of Internal Medicine and Gastroenterology, Katharinenhospital, Stuttgart, Germany; 3 Department for Interdisciplinary Endoscopy; Department of Internal Medicine I; and Department of Computational Neuroscience, University Hospital Hamburg - Eppendorf, Hamburg, Germany

## Abstract

**Background ** Reliable documentation is essential for maintaining quality standards in endoscopy; however, in clinical practice, report quality varies. We developed an artificial intelligence (AI)-based prototype for the measurement of withdrawal and intervention times, and automatic photodocumentation.

**Method**  A multiclass deep learning algorithm distinguishing different endoscopic image content was trained with 10 557 images (1300 examinations, nine centers, four processors). Consecutively, the algorithm was used to calculate withdrawal time (AI prediction) and extract relevant images. Validation was performed on 100 colonoscopy videos (five centers). The reported and AI-predicted withdrawal times were compared with video-based measurement; photodocumentation was compared for documented polypectomies.

**Results**  Video-based measurement in 100 colonoscopies revealed a median absolute difference of 2.0 minutes between the measured and reported withdrawal times, compared with 0.4 minutes for AI predictions. The original photodocumentation represented the cecum in 88 examinations compared with 98/100 examinations for the AI-generated documentation. For 39/104 polypectomies, the examiners’ photographs included the instrument, compared with 68 for the AI images. Lastly, we demonstrated real-time capability (10 colonoscopies).

**Conclusion ** Our AI system calculates withdrawal time, provides an image report, and is real-time ready. After further validation, the system may improve standardized reporting, while decreasing the workload created by routine documentation.

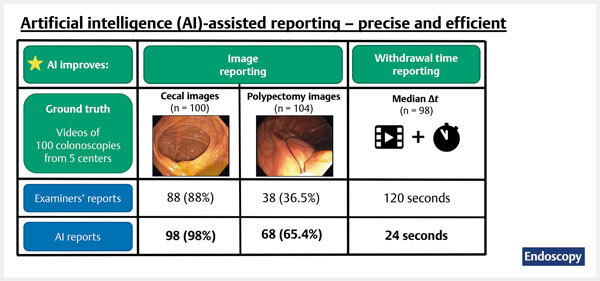

## Introduction

 As far back as 1992, Kuhn et al. 1 reported on the advantages of structured reporting in gastroenterology. While this improves the quality of patient care and research, it is not yet widely practiced owing to decreased flexibility and increased workload 2 3 4 . Along with other societies, the European Society of Gastroenterology (ESGE) released guidelines on screening colonoscopy performance measures in 2017 and reviewed their clinical application in 2021 5 6 7 8 . 

 The use of withdrawal time as a performance measure is based on an inverse correlation with the incidence of interval carcinomas 9 . The ESGE defines withdrawal time as “time spent on withdrawal of the endoscope from cecum to anal canal and inspection of the entire bowel mucosa at negative (no biopsy or therapy) screening or diagnostic colonoscopy,” calculated as the mean over 100 consecutive colonoscopies 5 . Currently it is common in clinical practice to determine the withdrawal time by basing the calculation on timestamps of a cecal and rectal image. In any case, there are no clear directions as to whether this image should be taken when reaching or when leaving the cecum. Additionally, there is no standardized practice to account for time not spent on mucosal inspection during withdrawal. The latter is especially important because studies frequently measure withdrawal time in examinations involving an endoscopic intervention. In this case, measurement is commonly performed with a stopwatch, which raises the question of whether withdrawal times measured in clinical practice and in studies are comparable. Furthermore, guidelines advise detailed photodocumentation as it allows re-evaluation at a later point, but the taking of photographs is purely dependent on the examiner and requires extra effort. 

Therefore, automatic detection of cecal intubation and withdrawal time, along with “backup” photodocumentation would improve standardized colonoscopy documentation and relieve endoscopists of the additional workload associated with this. In this study, we introduce a deep learning-based system for the automatic measurement of withdrawal time and photodocumentation of the cecum and any polypectomies.

## Methods

### Study design and aim

A frame-by-frame prediction artificial intelligence (AI) algorithm was developed to calculate the withdrawal and intervention times, and to extract an image series. The photodocumentation aimed to represent at least one landmark in the cecum, as well as any detected polyps and their resection. The system was evaluated on 100 prospectively recorded videos and applied in real time during 10 additional examinations. Reported and AI-predicted withdrawal times were then compared with video-based measurement. The information content of the examiners’ and the algorithms’ photodocumentation were then compared with the examination report.

### The label set

**Fig. 1:**
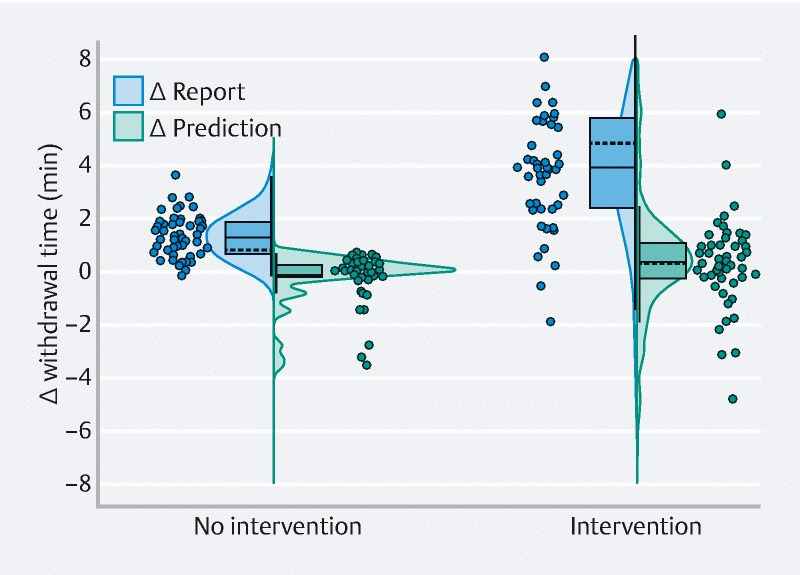
Comparison of the reported and AI-predicted withdrawal time difference from the measured withdrawal time. Withdrawal time difference (Δ) was calculated by subtraction of the measured time from either the reported time (blue) or AI-predicted time (red). Each curve represents a density plot of the data and is accompanied by a box plot of the data distribution. The dashed line within the density plot represents the mean; the solid line represents the median. Stars represent individual measurements (Δ Report No intervention, one measurement not shown as the value was ≤ 8 minutes; Δ Report Intervention, five measurements not shown as the values were > 8 minutes).

 For classification of single images, we defined labels for the cecum (“ileum,” “appendix” for appendiceal orifice, and “ileocecal valve”), for interventions (“polyp,” “chromoendoscopy” for virtual chromoendoscopy, “biopsy forceps,” “snare,” and “wound”), and for uninformative frames (“low quality” and “outside” for outside of the body) as shown in **Fig. 1 s** (see online-only Supplementary material). For video segmentation, labels representing various stages of an examination were defined (“outside,” “insertion,” “cecum,” “withdrawal,” and “intervention”). We labelled inspection, cleansing, and resection of polyps, as well as the subsequent post-polypectomy wound care as “intervention.” 

### Data selection

**Fig. 2:**
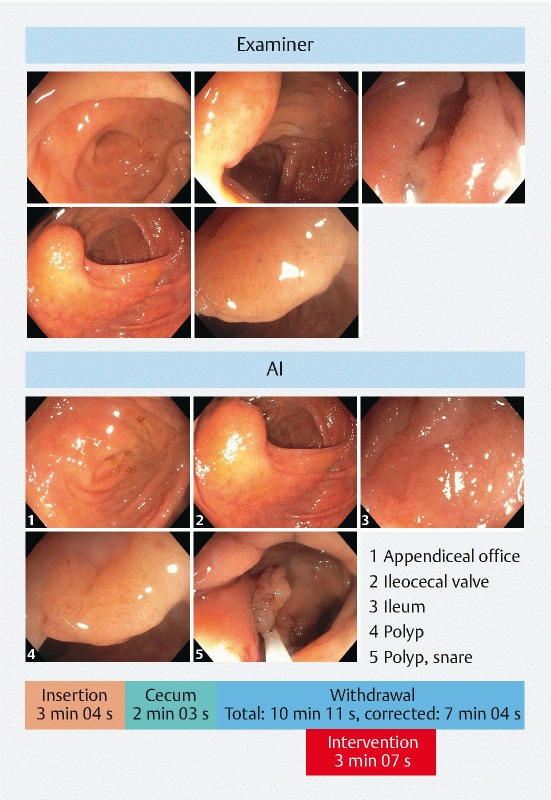
Example images showing an examiner’s documented images (top) and the AI-assistants documentation (bottom). Both reports contain an image of the appendiceal orifice, the ileocecal valve, the ileum, and the detected polyp, but the AI-generated report additionally displays the polyp during polypectomy. Furthermore, in the AI-generated report, a timeline displays the different phases of the intervention (insertion, cecum, withdrawal, and intervention).

 For training of the AI algorithm, 10 557 individual frames were collected from 1300 distinct colonoscopies in nine centers using four different processors (Olympus CV-170 and Evis Exera III CV-190, Olympus Europa SE & Co. KG, Hamburg, Germany; Pentax EPK-i7000, Pentax Europe GmbH, Hamburg, Germany; and Karl Storz Image1 S, Karl Storz SE & Co. KG, Tuttlingen, Germany). For each examination, a maximum of five images were selected per label to avoid data clustering. **Table 1 s** and **Fig. 2 s** summarize the number of annotated images per label, as well as the distribution between the training and in-training validation data. Examinations used during training were excluded from the subsequent test video selection. 

**Fig. 3:**
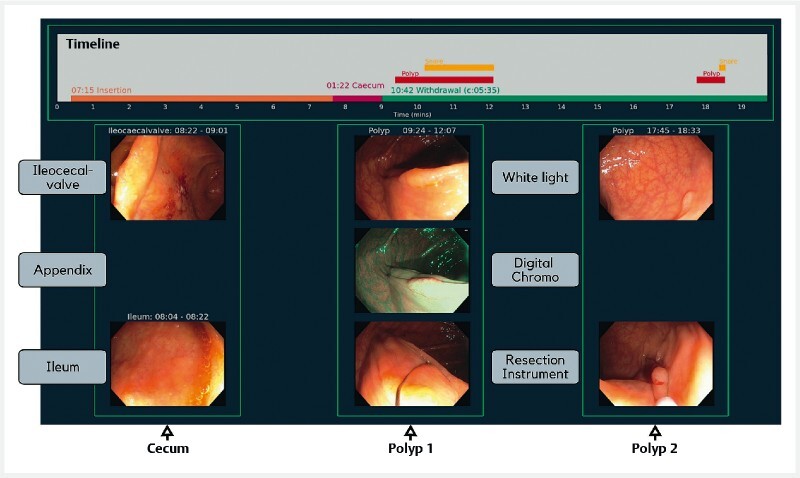
AI-generated colonoscopy summary after real-time application of the system in clinical practice. The video segmentation result is presented as color-coded timeline in the grey box above the images. The lowest bar in this box comprises of orange (insertion period), red (cecum period), and green segments (withdrawal time), labelled with their respective durations. Above this, red bars signify polyp sequences, while yellow bars represent the presence of a resection instrument, if applicable. The AI-selected images are displayed below the grey box: left column, landmarks of the cecal region; middle column, white-light inspection, narrow band imaging, and snare polypectomy of the first polyp sequence; right column, white-light inspection, snare polypectomy of the second polyp sequence.

 For testing of the video segmentation, full-length colonoscopy videos, with and without endoscopic intervention, were prospectively collected (five centers, four processors). The recorded examinations were screened chronologically (n = 100; 10 per group per center). Incomplete or corrupted videos (n = 76), and examinations of an already fully recruited test group (n = 97) were excluded. Examinations without a report (n = 69), with insufficient bowel preparation (Boston Bowel Preparation Scale [BBPS] < 6; n = 43), no cecal intubation (n = 5), inflammatory bowel disease, previously performed bowel surgery or radiation therapy were also excluded (n = 44). Furthermore, we excluded examinations in which a resection instrument was permanently visible during withdrawal (n = 42). The data collection process is summarized in **Fig. 3 s** . 

### Artificial intelligence model development

 The annotated images were split examination-wise into training (80 %), in-training validation (10 %), and after-training validation (10 %) datasets. With these images, a pretrained RegNetX800MF model from the *torchvision* library was fine-tuned for multilabel prediction 10 11 . The model training is described in detail in **Appendix 1 s** . Performance measures on the validation dataset are summarized in **Table 2 s** . 

### Withdrawal time

 The ESGE defines the withdrawal time as “Time spent on withdrawal of the endoscope from cecum to anal canal and inspection of the entire bowel mucosa […]” 5 . No statement regarding inspection of cecal mucosa and cleaning of the intestines exists. 

 We determined the “reported withdrawal time” via the report, if it was stated. Otherwise, timestamps of the last documented cecal and rectal images were used to calculate the reported time. Video-based “measurement of the withdrawal time” was determined by manually annotating the following video segments (shown in **Fig. 4 s** ):

1. *t*_insertion_  =  *t*_first cecum_ – *t*_enter body_
2. *t*_cecum inspection_  =  *t*_last cecum_ − *t*_first cecum_
3. *t*_withdrawal_  =  *t*_exit body_ – *t*_last cecum_
4. *t*_intervention_  = ∑ *t*_intervention end_ − *t*_intervention start_
5. *t*_cecum inspection corrected_  =  *t*_cecum inspection_ − *t*_intervention in cecum_
6. *t*_withdrawal corrected_  =  *t*_withdrawal_ – *t*_intervention during withdrawal_

Note: times were calculated separately for insertion, cecum inspection, and withdrawal.

 The “AI-predicted withdrawal time” was determined by post-processing the frame-by-frame predictions for each video resulting in a video segmentation corresponding to the annotations of the manual measurement ( **Appendix 1 s** ). 

### Image report generation

Images of each detected cecal region (ileum, ileocecal valve, and appendiceal orifice) and representative images of each detected polyp sequence were selected by the algorithm if available. Representative polyp images were defined as: (i) a white-light image, (ii) a digital chromoendoscopy image, and (iii) an image including the polyp and the resection instrument. Each selected image represented the frame with the highest confidence prediction value without prediction of an uninformative label (“low quality” or “outside”).

### Evaluation of generated image reports

Three board-certified gastroenterologists were randomly presented with either the examiner-created or AI-generated image report for 100 examinations. Examiners were blinded to the test group. The number of distinct polyps and each polyp’s resection method were annotated. Following a washout period of 6 weeks, the remaining images were presented to the examiners. Polyps and polypectomies described by less than two of the three examiners were disregarded.

### Implementation of real-time application

 For real-time application, the previously described EndoMind framework 12 was extended with the newly developed algorithm for multilabel classification and consecutive post-processing of the predictions. Real-time prediction is performed at a rate of 10 frames per second. 

### Ethical considerations

The study was approved by the local ethics committee responsible for each study center (Ethik-Kommission Landesärztekammer Baden-Württemberg [F-2021–047. F-2020–158], Ethik-Kommission Landesärztekammer Hessen [2021–2531], Ethik-Kommission der Landesärztekammer Rheinland-Pfalz [2021–15,955], and Ethik-Kommission University Hospital Würzburg [12/20, 20200114 04]). All procedures were in accordance with the Helsinki Declaration of 1964 and later versions. Signed informed consent was obtained from each patient prior to participation.

## Results

### Examination characteristics

We analyzed 10 examinations with endoscopic intervention and 10 without from each of the five centers with one endoscopist per center. All participating endoscopists have at least 10 years of experience. Overall, 75 % of the examinations were screening or surveillance colonoscopies (Table 3 s). In the 50 examinations with endoscopic intervention, a total of 104 polyps were detected and resected (Table 4 s). The majority (58 %) were sized 5–10 mm and were in the sigmoid (19 %) or ascending colon (18 %). Histopathology confirmed 70 polyps (67 %) to be adenomas or sessile serrated lesions.

### Withdrawal time measurement

 The algorithm could not determine a withdrawal time for two of the 100 examinations as no cecal landmark was detected. The reported withdrawal time diverged more than 20 % from the measurement in 33 of 50 examinations without endoscopic intervention and 44 of 50 examinations with intervention. For the AI predictions, this was the case in six of 50 and 18 of 48 of the examinations. The absolute time difference between the AI-predicted and measured withdrawal times was smaller than the difference between the reported and measured times in 44 of 50 cases in both groups. The median absolute differences between AI prediction and measurement were 0.25 minutes (no intervention) and 0.9 minutes (intervention), respectively, compared with 1.3 minutes and 3.9 minutes for the reported times. Fig. 1 demonstrates withdrawal time difference as a violin plot with individual measurements depicted as stars. The center-wise subanalysis is shown in **Fig. 5 s.**

### Evaluation of the AI-generated photodocumentation

 The AI-selected report images contained an identifiable image of the cecum in 98 examinations (98 %). Specifically, an image of the ileocecal valve was supplied from 85 examinations (85 %), of the appendiceal orifice in 79 %, and of the ileum in 49 %. Additionally, images of polyps, resection instruments (biopsy forceps or snare), and chromoendoscopy were included in the image reports. Table 1 details the specificity per label for images included in the generated photodocumentation. 

**Table 1:** Specificity of artificial intelligence-selected report images.

Label	Images containing label	AI-predicted images	Specificity 1
All	511	557	91.7 %
Appendiceal orifice	72	79	91.1 %
Biopsy forceps	5	10	50.0 %
Chromoendoscopy	12	14	85.7 %
Ileocecal valve	83	85	97.6 %
Ileum	48	49	98.0 %
Polyp	188	203	92.6 %
Snare	103	117	88.0 %

1 Annotation by a gastroenterologist revealed an overall specificity of > 91 % for the automatically selected images.

 The reports of 50 examinations with endoscopic intervention described a total of 104 polypectomies. Annotators identified 63/80 snare polypectomies (78.8 %) and 5/24 biopsy forceps polypectomies (20.8 %) in the AI-generated photodocumentation. In contrast, the endoscopists’ image series represented only 34/80 (42.5 %) and 5/24 polypectomies (20.8 %), respectively. Fig. 2 illustrates the AI- and examiner-selected image report of one examination. 

### Real-time application

 Lastly, the algorithm was successfully integrated into our previously described real-time polyp detection framework 12 . Fig. 3 shows the resulting video segmentation and photodocumentation generated after each examination. In all 10 colonoscopies, the system correctly identified a cecal landmark and the mean absolute difference between the measured and AI-calculated withdrawal times was 37 seconds (range 13–75 seconds). 

## Discussion

 Withdrawal time is an established performance parameter in clinical practice and research, yet its measurement is not standardized, with methods ranging from calculation by timestamps to manual stopwatch measurement. Furthermore, a prospective study revealed a drastic increase in withdrawal time and adenoma detection rate (ADR; 21.4 % to 36.0 %) when examiners knew that withdrawal time was being monitored 13 . 

 Based on these considerations, we developed a prototype to reliably determine withdrawal time and provide a backup image report to prevent documentation gaps. A novel feature is that our system processes the video signal to identify cecal intubation, polypectomies, and withdrawal time. In contrast, a previously published study relied on examiner-documented images for analysis 14 . Despite promising results in a research setting, a mean of 44.7 documented images per report were evaluated, which raises the question of whether clinical application would actually be feasible, given that the examiners in our study documented a mean of 8.6 images per examination (our AI system 5.5) during clinical routine. Other related works have monitored withdrawal speed 15 or quantified mucosal inspection 16 17 to enhance endoscopists’ intraprocedural performance. 

 While AI has recently progressed rapidly, the most researched applications in endoscopy aim to influence diagnostics or therapy; however, even in radiology, where experience with such systems is much greater than in gastroenterology, only a few reach clinical practice 18 . In this study, we demonstrate how AI may benefit clinical practice by measuring withdrawal time and providing “backup” photodocumentation. Instead of suggesting diagnoses or giving therapeutic advice, the system relieves endoscopists of the task of “measuring” withdrawal time and simultaneously lowers the risk of incomplete photodocumentation. We hypothesize that this could not only improve acceptance of structured reporting and application of AI, but also increase the report quality. 

While the prototype demonstrates functionality for four different processor signals, its generalizability should not be readily assumed, which is a limitation of our study. In particular, the recognition of instruments may vary if new instruments are used. Continuous performance monitoring and center-specific fine-tuning are however a necessity for all applied AI models as modalities can always change. In addition, we are not able to re-identify polyps.

In conclusion, this work proposes a paradigm-shift in medically applied AI: instead of competing with physicians, AI systems should first address the recommended comprehensive documentation of basic findings. In future, the skeleton of a colonoscopy report could be pre-generated, with the examiner then validating the content. Future research should continue to evaluate this approach and extend it to more report modalities, such as polyp classification or quantification of other pathologies.

## Tables 1 s–4 s, Figs. 1 s–5 s

Supplementary material Zusatzmaterial
