# Supplementary material for: Assisted documentation as a new focus for artificial intelligence in endoscopy: the precedent of reliable withdrawal time and image reporting
Source: Endoscopy. 2023 Aug 23;55(12):1118–23. doi: 10.1055/a-2122-1671 (PMC11321719; doi:10.1055/a-2122-1671)
Supplement: Supplementary file 1 — Supplementary material [file 22538supmat_10-1055-a-2122-1671.pdf]

Supplementary material

Supplementary material

Assisted documentation as new focus for artificial intelligence in endoscopy: The precedent of reliable withdrawal time and image reporting

Thomas J. Lux, Zita Saßmannshausen, Ioannis Kafetzis, Philip Sodmann, Katja Herold, Boban Sudarevic, Rüdiger Schmitz, Wolfram G. Zoller, Alexander Meining, Alexander Hann

Contents

	Page
Figure 1s	2
Table 1s	3
Figure 2s	3
Figure 3s	4
Appendix 1s	5
Table 2s	6
Figure 4s	6
Table 3s	7
Table 4s	7
Figure 5s	8

Lux T et al. Assisted documentation as a new focus for art… Endoscopy | © 2023. Thieme. All rights reserved.

Supplementary material

Figure 1s. Representative images of classification labels. 10 non mutually exclusive classes were annotated in colonoscopy images classes. Each class was selected to represent a category needed for further video segmentation. **(a)** Cecal landmarks: Ileum, ileocecal valve, and appendiceal orifice. **(b)** Polyp inspection: Polyp and chromoendoscopy. **(c)** Intervention-associated: Snare, biopsy forceps, wound. **(d)** Supporting labels: outside of body, and low quality.

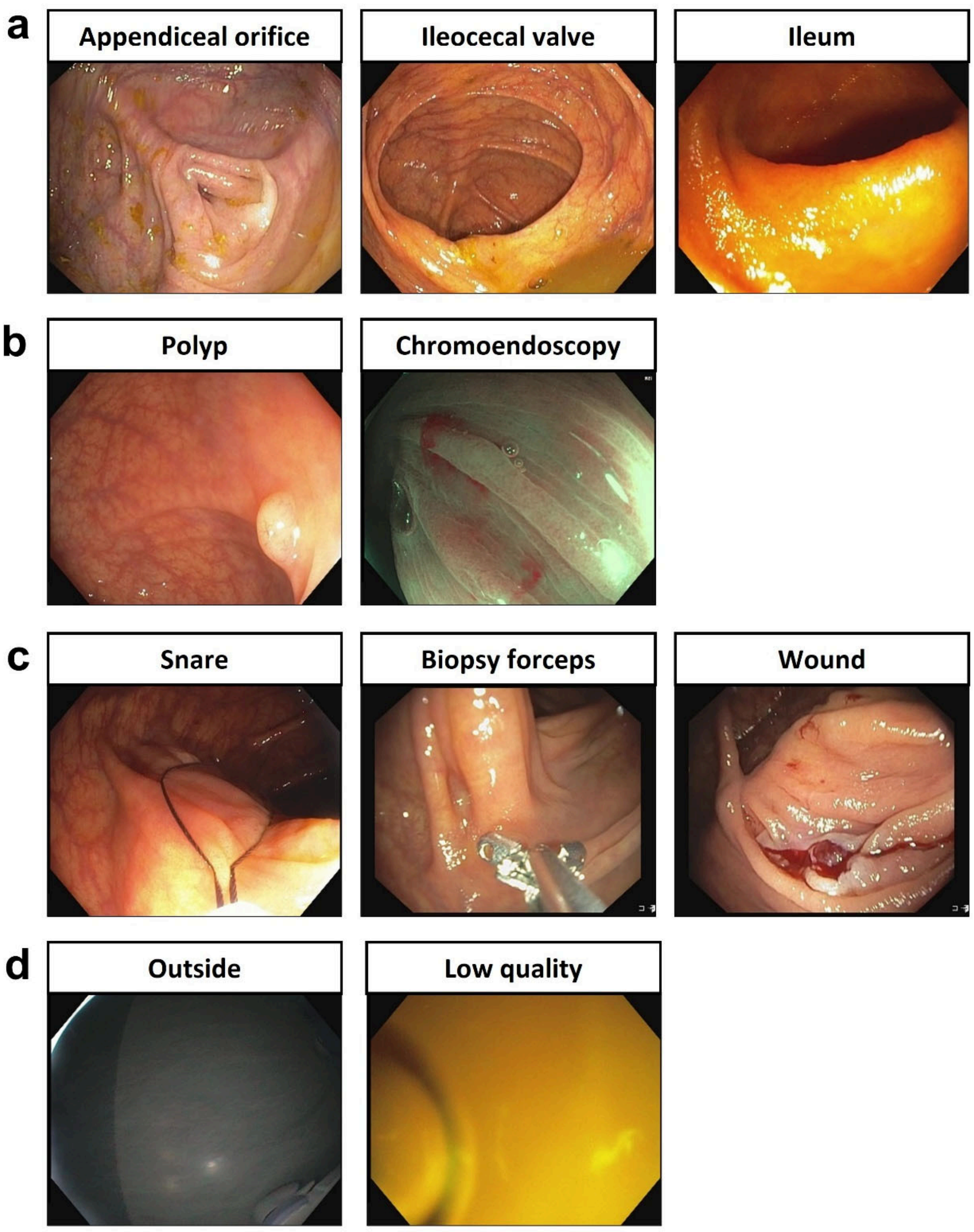

Lux T et al. Assisted documentation as a new focus for art... Endoscopy | © 2023. Thieme. All rights reserved.

Supplementary material

Table 1s. Number of training and validation images. Letters A to I represent individual centers.

Characteristic	***Train***	***Validation***	***Sum***
**Label**			
Appendix	917	107	1024
Biopsy forceps	440	51	491
Chromoendoscopy	325	33	358
Ileocecal valve	998	86	1084
Ileum	874	92	966
Low quality	1359	177	1536
Outside	984	100	1084
Polyp	1042	109	1151
Wound	247	30	277
Snare	824	104	928
**Center**			
A	2354	273	2627
B	937	117	1054
C	384	41	425
D	17	1	18
E	2979	339	3318
F	1359	130	1489
G	1130	121	1251
H	85	7	92
I	256	27	283

Figure 2s. Number of images annotated for the 10 non-exclusive labels. Bars indicate the number of images containing the corresponding label. The blue part of the bar indicates the fraction utilized for training while the red part indicates the fraction used for model validation during training. Images could contain no label. Once an image was assigned the train or validation set, the same image with different label could not be assigned to the other dataset. Co-incidences of labels in the same image are not visualized. In total 10,557 images were annotated.

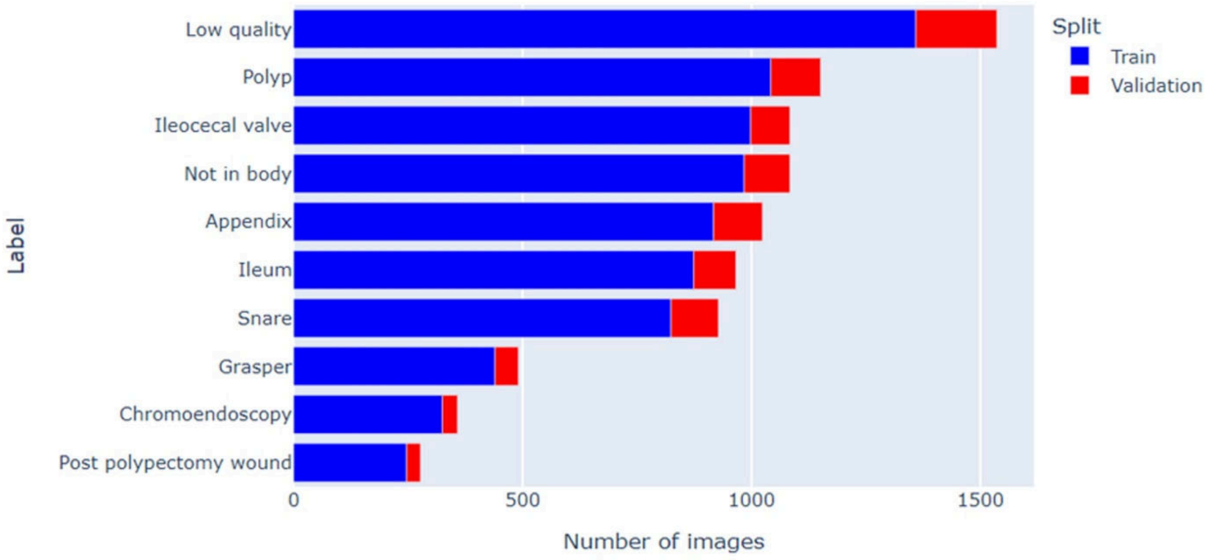

Lux T et al. Assisted documentation as a new focus for art... Endoscopy | © 2023. Thieme. All rights reserved.

Supplementary material

Figure 3s. Flowchart of the selection process for examinations. A total of 476 examinations were recorded to select 10 examinations with endoscopic intervention and 10 without for each of the 5 centers. Examinations with an invalid or incomplete video were excluded. Most common reasons were start of the recording after entering or before exiting the body. BBPS, Boston bowel preparation scale; IBD, inflammatory bowel disease.

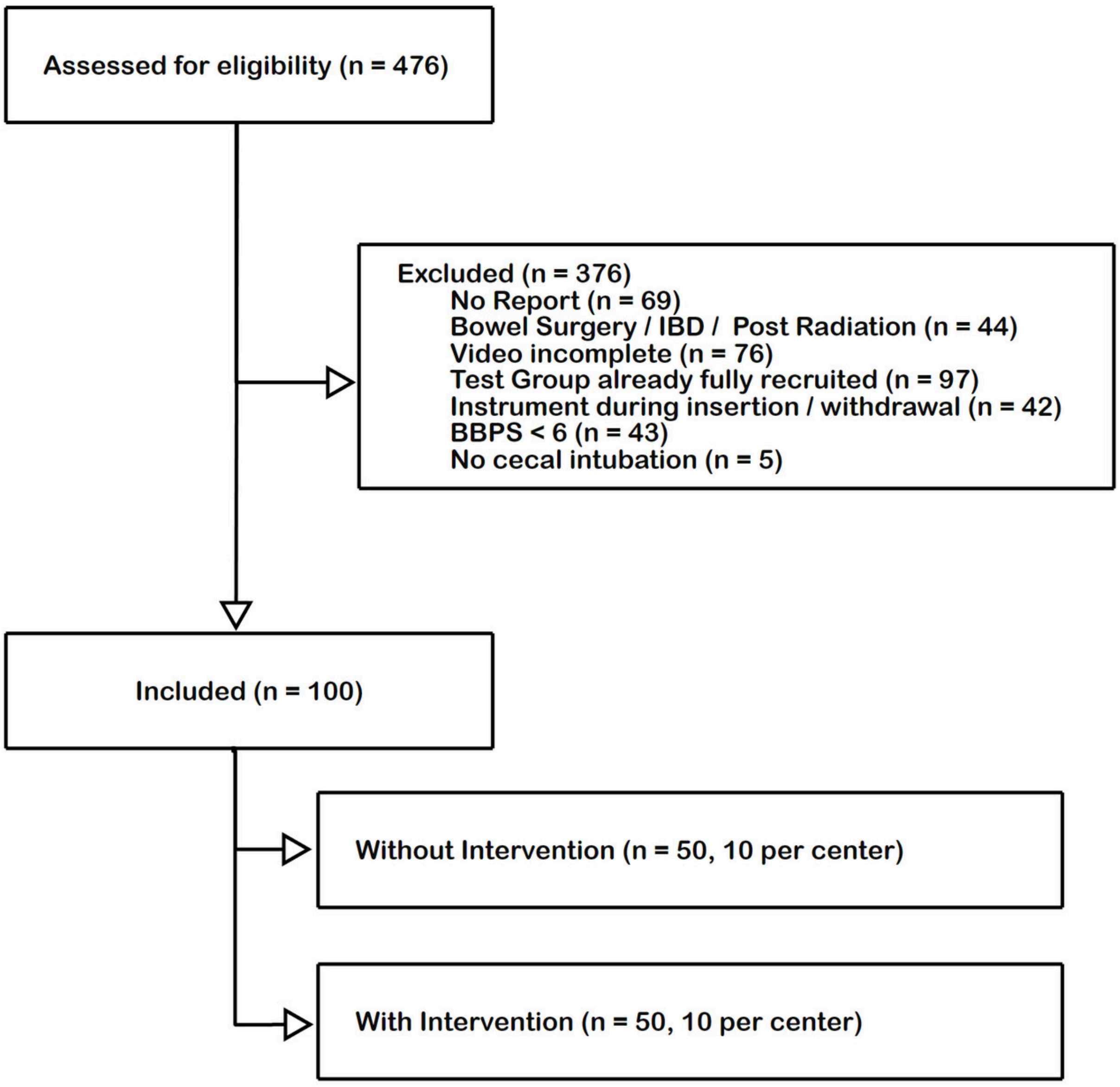

Lux T et al. Assisted documentation as a new focus for art… Endoscopy | © 2023. Thieme. All rights reserved.

Supplementary material

Appenidix 1s Method

AI-Development
Black borders of the images for training and annotation were cropped beforehand and zero-padded to yield square images. Images for training were augmented to diversify the dataset. This augmentation included random rotations by a multiple of 90°, mirroring the image along the horizontal or vertical axis, and appliance of distortion and blur filters.

Model training was performed using the *PyTorch Lightning* framework [1]. We utilized *PyTorch's* stochastic gradient descent with momentum implementation as optimizer (initial learning rate: 0.03, momentum = 0.5, weight decay = 0.003). The training target was to minimize binary cross entropy loss. During loss calculation, positive labels were weighted with a factor of 1.5. The learning rate during training was scheduled by *PyTorch's* cosine annealing with warm restarts implementation (restart every 20th epoch). Training was automatically stopped if the validation loss did not improve for 20 epochs of training.

Post-processing of framewise predictions
Prediction of each video resulted in a N_{Frame} by N_{Label} sized matrix containing the prediction values after applying a sigmoid function. A running mean including the frame-rate equivalent of 0.5 s before and after the current frame (e.g., +/- 25 frames at a recording rate of 50 frames per second). Then, predictions were binarized into true or false statements by applying a threshold of > 0.5 for true and <= 0.5 for false on each classes' prediction vector. Lastly, all other labels were set to false if a frame contained a positive prediction for *"low quality"* or *"outside"*.

Generating video sequences
The post processed matrices for each video were then transformed to video segmentations. Consecutive predictions of the same class were used to yield a list of start / stop tuples for each class. For noise reduction, sequences shorter than 1 s were disregarded as noise. Classes were then clustered into the categories annotated in the test data (outside, insertion, cecum, withdrawal, intervention). Here, a set of rules was applied on the sequences:

First, all subclasses of the intervention category were clustered. If segmentations had a time difference of less than 30 s, we assumed them belonging to the same intervention. In this case, the frames in between were added to this intervention sequence.

Subsequently, we merged the first and last appearances of appendix, ileum and / or ileocecal valve sequences to evaluate time spent in the cecum. Time from start of the examination (defined as start of the video or entering the body) to the first cecal landmark was assumed as insertion time. Time from the last detected cecal landmark until end of the video or last exit of the body was determined as withdrawal time. To minimize disturbance of our calculation by brief false positive sequences of cecal landmarks, the last detected landmark sequence was disregarded if the following criteria were met: 1. Withdrawal time is below 100 s; 2. Difference between the last detected and the preceding cecal landmark is greater than 120 s.

Lux T et al. Assisted documentation as a new focus for art... Endoscopy | © 2023. Thieme. All rights reserved.

Supplementary material

Table 2s. Best model performance on the validation dataset during model training. Precision: Fraction of correct predictions among all predictions of a given label; Recall: Fraction of correctly identified images among all images with a given label; F1: Mean value of precision and recall.

Label	F1 (%)	Precision (%)	Recall (%)
**Appendix**	57.7	61.7	54.2
**Biopsy forceps**	67.1	52.8	92.2
**Chromoendoscopy**	93.9	93.9	93.9
**Ileocecal valve**	69.4	69.0	69.8
**Ileum**	80.0	94.1	69.6
**Low quality**	85.3	91.0	80.2
**Outside**	98.5	99.0	98.0
**Polyp**	64.4	68.8	60.5
**Snare**	86.3	85.1	87.5
**Wound**	40.8	52.6	33.3

Figure 4s. Schematic overview of different resulting time span classes for withdrawal time calculation. The arrow represents the timeline of a colonoscopy. Boxes located on top of the timeline signify the occurrence of the events labeled in the box (grey: video start / stop; blue: entering / exiting the body; green: entering and exiting the cecum / terminal ileum; red: start / stop of endoscopic interventions / cleaning). Times calculated with timestamps of these events are visualized as green bars below the timeline-arrow.

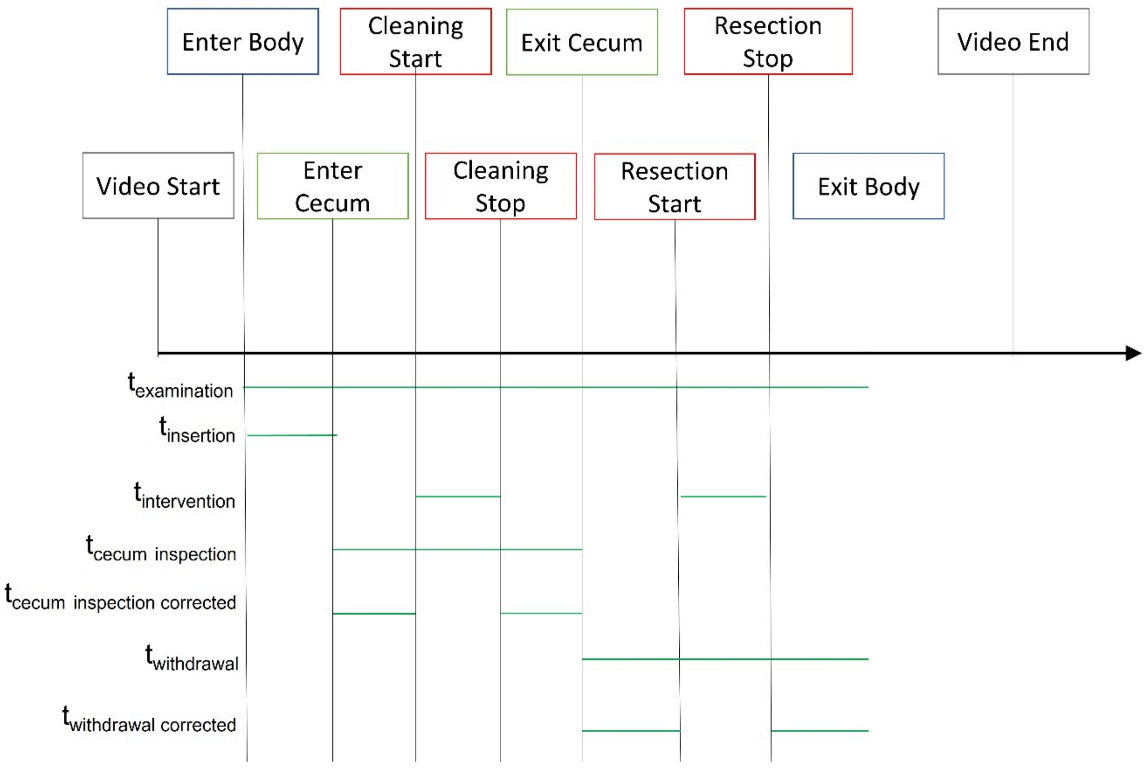

Lux T et al. Assisted documentation as a new focus for art… Endoscopy | © 2023. Thieme. All rights reserved.

Supplementary material

Table 3s. Characteristics of the 100 examinations used as test data. CI, confidence interval; BBPS Boston Bowel Preparation Scale

Characteristic	Value
**Gender**	
Female	50
Male	50
**Age, median [95-CI]**	59 [57-62]
**Indication**	
Screening	64
Surveillance	9
Symptomatic	16
Unknown	11
**BBPS, median [95-CI]**	8 [8-9]

Table 4s. Characteristics of the 104 detected polyps.

Characteristic	Count [%]
**Morphology**	
Sessile	68 [65.4]
Flat	31 [29.8]
Pedunculated	5 [4.8]
**Size**	
< 5 mm	32 [30.8]
5–10 mm	61 [58.7]
>10–20 mm	7 [6.7]
> 20 mm	4 [3.8]
**Histology**	
Tubular adenoma	47 [45.2]
Non-adenoma	24 [23.1]
Sessile serrated lesion	18 [17.3]
Tubulovillous adenoma	5 [4.8]
Unknown	9 [8.7]
Carcinoma	1 [1.0]
**Resection / Biopsy method**	
Snare	80 [76.9]
Biopsy forceps	24 [23.1]

Lux T et al. Assisted documentation as a new focus for art... Endoscopy | © 2023. Thieme. All rights reserved.

Supplementary material

Figure 5s. Center-wise comparison of reported and predicted withdrawal time difference to measurement. Withdrawal time difference (Δ) was calculated by subtraction of the measured time from either the reported (blue) or predicted (red) time. Each curve represents a density plot of the data and is accompanied by a box plot of the data distribution. The dashed line within the density plot represents the mean whereas the solid line represents the data's median value. Stars represent individual measurements**.**

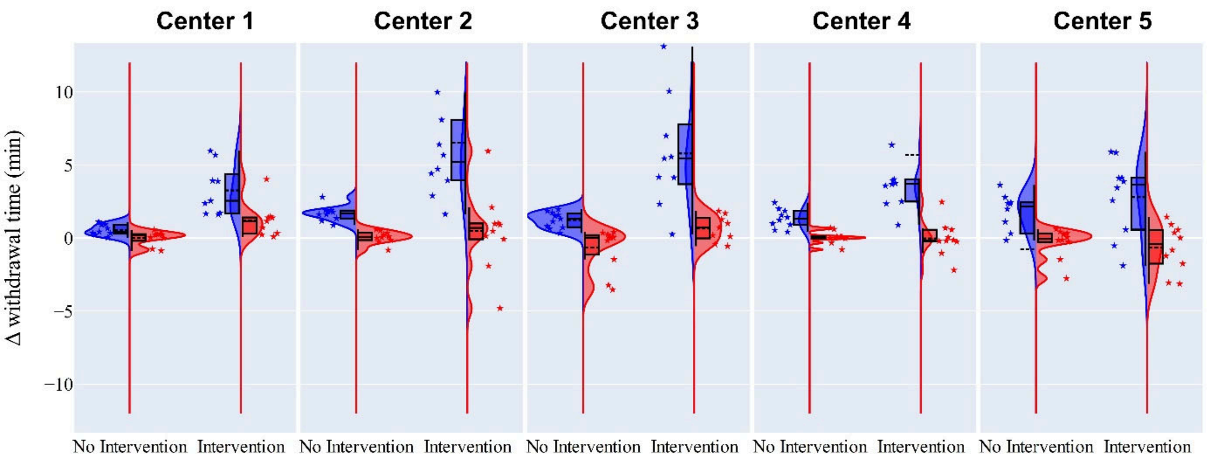

SUPPLEMENTARY REFERENCE

1 Falcon W, The PyTorch Lightning team. PyTorch Lightning. 2019. doi:10.5281/zenodo.3828935

Lux T et al. Assisted documentation as a new focus for art… Endoscopy | © 2023. Thieme. All rights reserved.
